# Safety assessment of sapropterin dihydrochloride: real-world adverse event analysis based on the FDA adverse event reporting system (FAERS)

**DOI:** 10.3389/fphar.2024.1486597

**Published:** 2024-10-25

**Authors:** Jiahong Zhong, Xihui Yu, Zhuomiao Lin

**Affiliations:** ^1^ Department of Clinical Pharmacy, Meizhou People’s Hospital (Huangtang Hospital), Meizhou, Guangdong, China; ^2^ Department of Pharmacy, The Second Affiliated Hospital of Shantou University Medical College, Shantou, Guangdong, China

**Keywords:** sapropterin, FAERS, adverse events, phenylketonuria, hyperphenylalaninemia

## Abstract

**Objective:**

Sapropterin dihydrochloride is the first drug for the therapy of phenylketonuria, which is a rare disease that occurs one of 10,000–15,000 newborns. As a result, detailed and comprehensive reports on the safety of sapropterin in large, real-world populations are required. The purpose of this study is to undertake a complete analysis of sapropterin’s adverse events (AEs) using the FDA Adverse Event Reporting System (FAERS) database.

**Methods:**

We retrieved reports of adverse events with sapropterin as the principal suspect from FAERS between the first quarter of 2008 and the first quarter of 2024. The Reporting Odds Ratio (ROR), Proportional Reporting Ratio (PRR), and Bayesian Confidence Propagation Neural Network (BCPNN) were utilized to detect AE signals.

**Results:**

The study collected 4,953 suspected AE cases from the FAERS database, with sapropterin as the major suspect. A total of 130 positive signals were obtained utilizing the ROR, PRP, and BCPNN. The FAERS database revealed that common clinical AEs of sapropterin included vomiting, upper respiratory infection, rhinorrhea, and a reduction in amino acid concentrations. Furthermore, we detected probable unexpected adverse events (AEs) using disproportionality analysis, including gastroesophageal reflux disease, flatulence, influenza, ear infection, viral infection, pharyngitis streptococcal, spontaneous abortion, and nephrolithiasis.

**Conclusion:**

By analyzing huge amounts of real-world data from the FAERS database, we found potential novel AEs of sapropterin using disproportionate analysis. It is advantageous for healthcare professionals and pharmacists to focus on efficiently managing sapropterin’s high-risk adverse events, improving drug levels in clinical settings, and ensuring patient medication safety.

## 1 Introduction

Phenylketonuria (PKU) is a congenital metabolic condition caused by phenylalanine hydroxylase deficiency, resulting in elevated phenylalanine (Phe) levels (hyperphenylalaninemia, HPA) of varying severity (Blau et al., 2010; Kure and Shintaku, 2019). A tiny percentage of HPA patients (3%) have tetrahydrobiopterin (BH4) insufficiency, which is characterized by a congenital absence of the enzymes required for BH4 synthesis (Blau et al., 2010). Without treatment, HPA/PKU may result in cognitive damage and, in the case of a severe phenotypic with substantial HPA, profound and irreversible mental handicap (Levy et al., 2007; Blau et al., 2010; Opladen et al., 2020). PKU prevalence is highest in white or East Asian populations (about 1:10,000–15,000 live births) (van Spronsen et al., 2021). Among 1,07,078,115 neonates examined for HPA in China, 380 with BH4D were identified, corresponding to a total prevalence of 3.8 per 1,000,000 live births. The prevalence is slightly higher than that recorded for other nations, and it varies significantly across China (Yuan et al., 2021). High quantities of phenylalanine in embryonic life, such as those found in maternal PKU, can cause microcephaly, neuronal loss, and corpus callosum hypoplasia. Elevated phenylalanine levels in the first few years of life can result in acquired microcephaly, severe cognitive impairment, and epilepsy, most likely due to impaired synaptogenesis (Rovelli and Longo, 2023). Lowering the plasma phenylalanine level avoids intellectual impairment, whereas keeping levels in the therapeutic range of 120–360 µmol/L is leads to positive outcomes for individuals and their pregnancies (Lichter-Konecki and Vockley, 2019).

Sapropterin dihydrochloride (Kuvan), hereafter referred to as sapropterin, is a synthetic formulation of the active 6R-isomer of BH4, a naturally occurring cofactor for phenylalanine hydroxylase. It was first licensed as a therapy for tetrahydrobiopterin deficiency in Japan in 1992, and was then approved as a treatment for a tetrahydrobiopterin-responsive hyperphenylalaninemia in 2007 and 2008, in the United States and Japan, respectively (Shintaku et al., 2021). In the European Union, sapropterin is authorized for the treatment of hyperphenylalaninaemia in patients ≥4 years old with tetrahydrobiopterin-responsive phenylketonuria (PKU), as well as adults and children with tetrahydrobiopterin deficiency who are receptive to such treatment (Sanford and Keating, 2009). Sapropterin is given to patients with tetrahydrobiopterin deficiency to replenish endogenous tetrahydrobiopterin levels (Sanford and Keating, 2009). The European Medicines Agency has expanded the indication for sapropterin from the treatment of BH_4_-responsive PKU in adults and children aged ≥4 years and in all BH_4_-deficient adults and children with BH_4_-responsive PKU <4 years old, for whom the previous standard of care was a phenylalanine-restricted diet (Blau, 2013; Muntau et al., 2017). A longitudinal follow-up study of all patients with BH_4_-responsive PAH deficiency in Japan found that sapropterin was an effective treatment for keeping blood phenylalanine levels within the desirable range and was safe for Japanese patients with BH4-responsive PAH deficiency (Shintaku and Ohura, 2014). However, due to the small number of patients treated with sapropterin at the time of licensure, the safety of the drug has piqued the interest of both patients and healthcare professionals. A SPARK open-label, multicentre, randomized phase III b trial discovered widespread adverse events (AEs) of sapropterin including amino acid concentration decrease, rhinitis, and vomiting (Muntau et al., 2017). Further research is needed to investigate the potential AE signals of sapropterin in real-world situations, identify rare and severe AEs linked with this medicine, and encourage the safe use of sapropterin among PKU patients.

Because of the constraints of clinical trials, certain delayed and infrequent adverse events (AEs) may go unreported, although post-market data analysis can enhance safety information. Although certain AEs have been documented in clinical studies, PKU is a rare inherited metabolic disease with a global incidence of only 1:23,930. The reports and sample sizes of clinical trials of sapropterin were scarce, which was easy to be prejudiced in the outcomes. Some rare AEs of sapropterin are easily overlooked, and long-term follow-up for safety is insufficient. In order to identify unreported adverse reactions and verify the safety of sapropterin reported in clinical trials, the AEs of sapropterin were analyzed utilizing FDA Adverse Event Reporting System (FAERS) database. FAERS is a sophisticated tool for analyzing adverse drug-related occurrences. The purpose of this study was to investigate sapropterin-related adverse events and patient characteristics in the FAERS database. The study might be a significant resource for future clinical use and improve drug safety for people with PKU.

## 2 Materials and methods

### 2.1 Data source and collection

We conducted a retrospective pharmacovigilance study on adverse events (AEs) of sapropterin dihydrochloride tablets based on the FAERS database, which is a publicly available database of safety reports submitted by consumers, pharmacists, and pharmaceutical companies around the world since 2004. AEs were collected from the first quarter of 2008 to the first quarter of 2024 based on the launch date of sapropterin dihydrochloride tablets.

### 2.2 Data processing

**FIGURE 1:**
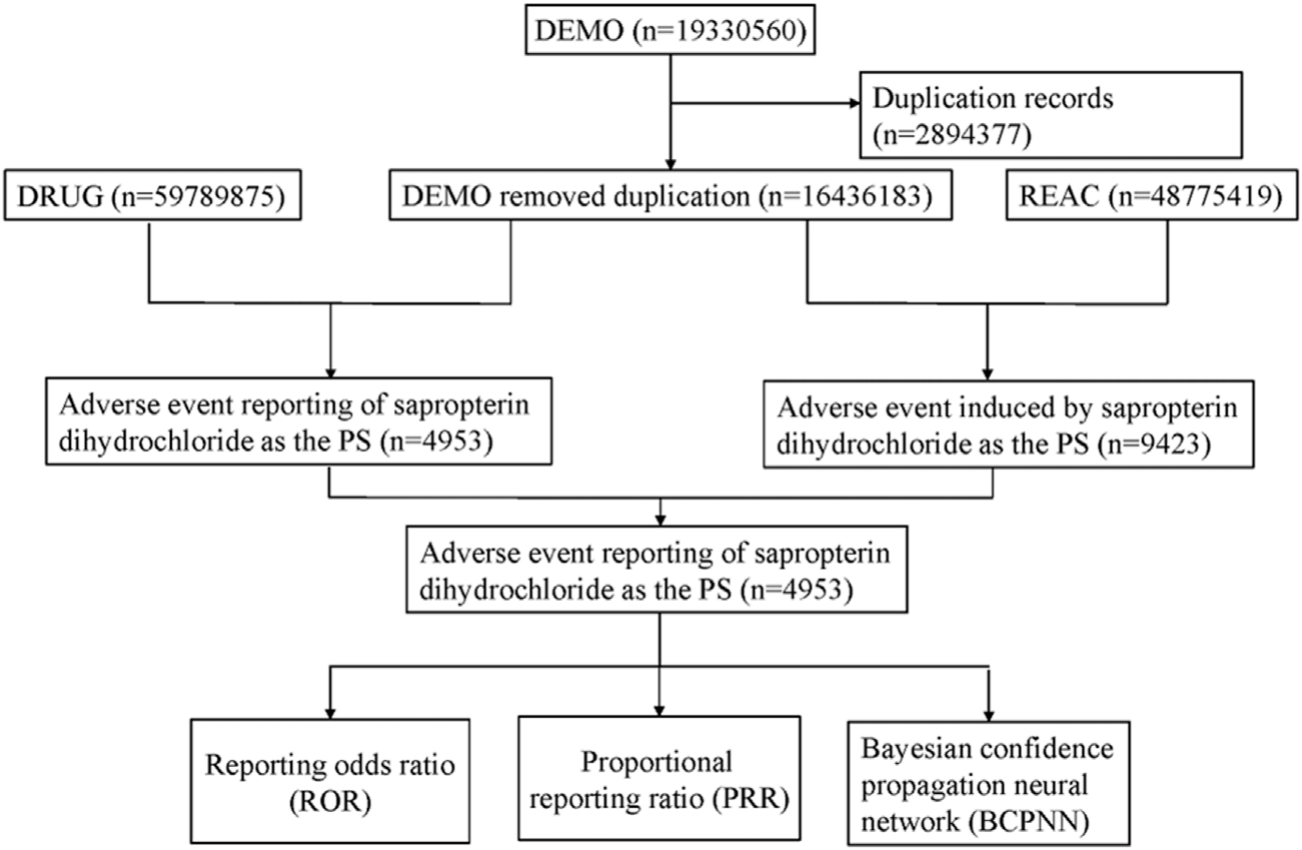
Flow diagram of the study (DEMO, demographic and administrative information; DRUG drug Information; REAC, preferred terminology for adverse drug reactions; PS, primary suspect drug).

We wrote the downloaded XML data package into RStudio and cleaned the data following the recommendations from the FDA. We used the Medical Subject Headings (MeSH) thesaurus (https://www.ncbi.nlm.nih.gov/mesh) to search all the drug nomenclature of sapropterin including medication brand, trade names, generic names and non-proprietary names. The generic name “sapropterin dihydrochloride” and trade name “KUVAN” were used to identify related reports. The study screened reports within the database that contained any of the following trade or generic names of the drugs: “KUVAN,” “KUVAN 100 MG TABLET SOL,” “KUVAN SAPROPTERIN DIHYDROCHLORIDE,” “KUVAN SAPROPTERID DIHYDROCHLORIDE 100 MG,” “KUVAN SAPROPERTIN DIHYDROCHLORIDE 100 MG 100 MG,” “TETRAHYDROBIOPTERIN,” “TETRAHYDROBIOPTERIN 100 MG 100 MG,” “TETRAHYDROBIOPTERIN TETRAHYDROBIOPTERIN,” “SAPROPTERIN DIHYDROCHLORIDE,” “6R BH4 SAPROPTERIN DIHYDROCHLORIDE TABLET 100MG.” Only the reports of sapropterin dihydrochloride with role code as the primary suspected drug were chosen for analysis. The preferred term (PT) from the Medical Dictionary for Regulatory Activities (MedDRA) should be utilized for standardized encoding when referring to AEs names in the reports. We also conducted SOC classification for all AEs. Duplicate reporting occurs when the same report is submitted by the consumer and the sponsor. Following the FDA-recommended method for removing duplicate reports, we selected the PRIMARYID, CASEID, and FDA_DT fields from the DEMO table. We sorted by CASEID, FDA_DT, and then PRIMARYID. For reports with the same CASEID, we retained the one with the largest FDA_DT value because the largest value means that its reporting date is the most recent. Secondly, for reports where both CASEID and FDA_DT are the same, we retain the one with the largest PRIMARYID value. The cleaned and standardized data was compiled into a final dataset, which is ready for analysis. This dataset included only those reports where sapropterin dihydrochloride was listed as the primary suspected drug (PS), aligning with our study’s focus. During the study period, 19,330,560 reports related to sapropterin dihydrochloride were obtained from the FAERS database. 16,436,183 reports remained after excluding duplicates, and 4,953 AEs were associated with sapropterin dihydrochloride (Figure 1). All AEs reports for sapropterin dihydrochloride were analyzed at the System Organ Class (SOC) and PT levels.

### 2.3 Statistical analysis

Methods with high sensitivity can detect more potential AEs and reduce the likelihood of missing true signals, while methods with high specificity can diminish the proportion of false positive signals (Jiao et al., 2024). For example, Reporting Odds Ratio (ROR) and Proportional Reporting Ratio (PRR) have high sensitivity and low specificity, whereas Bayesian Confidence Propagation Neural Network (BCPNN) and multi-item gamma Poisson shrinker (MGPS) high specificity and low sensitivity. ROR and PRR methods were selected in order to mine more ignored adverse reaction signals in this paper. At the same time, in order to avoid the misleading of false positive signals, we chose a highly specific method that was BCPNN rather than other specific methods because it was most suitable when the level of confounding increases and/or the effect sizes become larger. In the data mining process, we used ROR, PRR and BCPNN to reduce the bias of false-positive results caused by one method. The equations and criteria for the three algorithms are described in Table 1. AEs signals that satisfied all three algorithm criteria were considered significant signals. Data were analyzed using Microsoft Excel 2021 and R 4.3.0.

**TABLE 1:** Calculation formula and standard of signal detection.

Algorithm	Calculation formula	Criterion
ROR	$\text{ROR}=\frac{a/c}{b/d}=\frac{ad}{bc}$	a ≥ 3
	$95\%\text{CI}=e^{lnROR\pm 1.96\sqrt{\frac{1}{a}+\frac{1}{b}+\frac{1}{c}+\frac{1}{d}}}$	95% CI (lower limit) > 1
PRR	$\text{PRR}=\frac{a/\left(a+b\right)}{c/\left(c+d\right)}$	a ≥ 3, PRR ≥ 2
	$\chi^{2}=\frac{{\left(ad-bc\right)}^{2}\left(a+b+c+d\right)}{\left(a+b\right)\left(a+c\right)\left(c+d\right)\left(b+d\right)}$	χ2 ≥ 4
BCPNN	$\text{IC}=\log_{2}\frac{a\left(a+b+c+d\right)}{\left(a+b\right)\left(a+c\right)}$ $E\left(IC\right)=\log_{2}\frac{\left(a+\gamma_{11}\right)\left(N+\alpha \right)\left(N+\beta \right)}{\left(N+\gamma \right)\left(a+b+\alpha_{1}\right)\left(a+c+\beta_{1}\right)}$ $V\left(IC\right)=\frac{1}{{\left(\ln2\right)}^{2}}\left[\frac{N-a+\gamma -\gamma_{11}}{\left(a+\gamma_{11}\right)\left(N+1+\gamma \right)}+\frac{N-a-b+\alpha -\alpha_{1}}{\left(a+b+\alpha_{1}\right)\left(N+1+\alpha \right)}+\frac{N-a-c+\beta -\beta_{1}}{\left(a+c+\beta_{1}\right)\left(N+1+\beta \right)}\right]$ $\gamma =\gamma_{11}\frac{\left(N+\alpha \right)\left(N+\beta \right)}{\left(a+b+\alpha_{1}\right)\left(a+c+\beta_{1}\right)}$ $95\%CI=E\left(IC\right)\pm 1.96\sqrt{V\left(IC\right)}$ Where α = α1 + α2, β = β1 + β2, N = a + b + c + d, and the value of α1, α2, β1, β2 and γ11 were defined as 1.	The lower limit of 95%CI (IC025) > 0

Abbreviations: a, number of reports containing both the target drug and target adverse drug reaction; b, number of reports containing other adverse drug reaction of the target drug; c, number of reports containing the target adverse drug reaction of other drugs; d, number of reports containing other drugs and other adverse drug reactions. 95% CI, 95% confidence interval; IC, information component; IC025, the lower limit of 95% CI, of the IC; ROR, reporting odds ratio.

## 3 Results

### 3.1 General characteristics

From the first quarter of 2008 to the first quarter of 2024, a total case of 4,953 cases of sapropterin dihydrochloride were found om the FAERS database. Clinical characteristics of AEs to sapropterin dihydrochloride are shown in Table 2. In terms of gender, approximately 52.55% patients were female and 33.60% were male. In addition to the majority of unknown ages, the vast majority of reports focused on the age group under 18 years (19.26%), followed by the age group of 18–44 years (18.88%). The vast majority of reports were provided by consumer (79.37%). The top country reported was the United States (94.71%). Excluding unknown outcome, Other serious (important medical event) get the most reports.

**TABLE 2:** Characteristics of reports associated with sapropterin dihydrochloride.

Factors	Number of events (%)
Case reports	4,953 (100)
Gender	
Female	2,603 (52.55)
Male	1,664 (33.60)
Unknown	686 (13.85)
Age (year)	
<18	954 (19.26)
18–44	935 (18.88)
45–64	238 (4.81)
65–74	18 (0.36)
≥75	2 (0.04)
Unknown	2,806 (56.65)
Reporter	
Consumer	3,931 (79.37)
Health professional	449 (9.07)
Physician	237 (4.78)
Other health professional	197 (3.98)
Pharmacist	59 (1.19)
Unknown	80 (1.62)
Reporter country	
United States	4,691 (94.71)
Canada	80 (1.62)
India	45 (0.91)
Germany	21 (0.42)
Others	116 (2.34)
Outcome	
Hospitalization (initial or prolonged)	323 (6.52)
Death	59 (1.19)
Congenital anomaly	20 (0.40)
Life threatening	18 (0.36)
Disability	8 (0.16)
Other serious (important medical event)	397 (8.02)
Unknown	4,128 (83.34)
AE occurrence time - Medication date (days)	
0–30	360 (35.75)
31–60	80 (7.94)
61–90	55 (5.46)
91–120	44 (4.37)
121–150	22 (2.18)
151–180	18 (1.79)
181–360	103 (10.23)
>360	325 (32.27)

### 3.2 Signal detection

The signal strength of sapropterin dihydrochloride at the System Organ Class (SOC) level is shown at Table 3. After conducting an analysis, we have identified a total of 23 organ systems that are affected by adverse drug reactions caused by the sapropterin dihydrochloride. The most frequently reported SOC was gastrointestinal disorders.

**TABLE 3:** The signal strength of sapropterin dihydrochloride at the system organ class (SOC) level.

System organ class (SOC)	Case reports	ROR (95% CI)	PRR (χ^2^)	IC (IC025)
Gastrointestinal disorders	1,714	2.39 (2.27–2.52)	2.14 (1137.97)	1.1 (−0.57)
General disorders and administration site conditions	1,396	0.81 (0.77–0.86)	0.84 (52.6)	−0.25 (−1.92)
Nervous system disorders	1,093	1.43 (1.35–1.53)	1.38 (126.16)	0.47 (−1.2)
Psychiatric disorders	1,046	2.09 (1.96–2.23)	1.97 (528.66)	0.98 (−0.69)
Infections and infestations	737	1.52 (1.41–1.64)	1.48 (120.93)	0.57 (−1.1)
Injury, poisoning and procedural complications	727	0.72 (0.66–0.77)	0.74 (75.53)	−0.44 (−2.1)
Respiratory, thoracic and mediastinal disorders	564	1.3 (1.19–1.41)	1.28 (35.98)	0.35 (−1.31)
Skin and subcutaneous tissue disorders	311	0.59 (0.53–0.66)	0.61 (83.57)	−0.72 (−2.39)
Metabolism and nutrition disorders	217	1.09 (0.95–1.24)	1.08 (1.47)	0.12 (−1.55)
Musculoskeletal and connective tissue disorders	209	0.41 (0.35–0.46)	0.42 (178.37)	−1.26 (−2.92)
Pregnancy, puerperium and perinatal conditions	202	5.2 (4.53–5.98)	5.11 (670.38)	2.35 (0.69)
Renal and urinary disorders	69	0.38 (0.3–0.48)	0.38 (70.44)	−1.39 (−3.06)
Immune system disorders	59	0.56 (0.43–0.72)	0.56 (20.61)	−0.84 (−2.5)
Eye disorders	58	0.31 (0.24–0.4)	0.31 (89.42)	−1.68 (−3.34)
Congenital, familial and genetic disorders	56	1.9 (1.46–2.47)	1.89 (23.72)	0.92 (−0.74)
Vascular disorders	56	0.28 (0.21–0.36)	0.28 (104.47)	−1.83 (−3.49)
Cardiac disorders	47	0.2 (0.15–0.26)	0.2 (152.96)	−2.31 (−3.98)
Hepatobiliary disorders	42	0.5 (0.37–0.68)	0.51 (20.39)	−0.98 (−2.65)
Neoplasms benign, malignant and unspecified (incl cysts and polyps)	38	0.14 (0.1–0.2)	0.15 (193.56)	−2.77 (−4.43)
Ear and labyrinth disorders	36	0.88 (0.63–1.21)	0.88 (0.63)	−0.19 (−1.86)
Reproductive system and breast disorders	34	0.4 (0.29–0.56)	0.4 (30.03)	−1.3 (−2.97)
Blood and lymphatic system disorders	22	0.14 (0.09–0.21)	0.14 (117.09)	−2.83 (−4.49)
Endocrine disorders	12	0.5 (0.28–0.88)	0.5 (5.93)	−0.99 (−2.66)

Based on the frequency of occurrence, the top 20 PTs were listed at Table 4. Sorted by frequency (Table 4), the top 10 PTs with the highest number of reports are as follows: “Vomiting,” “Abdominal pain upper,” “Abdominal discomfort,” “Nasopharyngitis,” “Oropharyngeal pain,” “Dyspepsia,” “Gastrooesophageal reflux disease,” “Rhinorrhoea,” “Influenza” and “Abortion spontaneous.” We ranked the PTs that satisfied the criteria of the three techniques based on their signal intensity (ROR value) in descending order (Supplementary Table S1). It was observed that “Teething,” “Growth accelerated,” “Hand-foot-and-mouth disease,” “Dermatitis diaper” and “Hyperemesis gravidarum” ranked in the top five, with high level of signal intensity and not documented in the prescribing information. The relative proportion of the different PT to the total patients (N = 4,953) was also presented in Table 4.

**TABLE 4:** The top 20 PTs ranked by report numbers.

SOC	PTs	Case reports (%)	ROR (95% CI)	PRR (χ^2^)	IC (IC025)
Gastrointestinal disorders	Vomiting	264 (5.33)	3.87 (3.43–4.38)	3.79 (546.64)	1.92 (0.26)
Gastrointestinal disorders	Abdominal pain upper	187 (3.78)	6.08 (5.26–7.02)	5.98 (776.45)	2.58 (0.91)
Gastrointestinal disorders	Abdominal discomfort	151 (3.05)	5.73 (4.88–6.73)	5.65 (579.2)	2.5 (0.83)
Infections and infestations	Nasopharyngitis	124 (2.50)	4.37 (3.66–5.22)	4.32 (317.51)	2.11 (0.45)
Respiratory, thoracic and mediastinal disorders	Oropharyngeal pain	76 (1.53)	5.05 (4.03–6.33)	5.02 (244.46)	2.33 (0.66)
Gastrointestinal disorders	Dyspepsia	73 (1.47)	5.04 (4–6.34)	5.01 (234.21)	2.32 (0.66)
Gastrointestinal disorders	Gastrooesophageal reflux disease	72 (1.45)	5.96 (4.72–7.51)	5.92 (294.25)	2.56 (0.9)
Respiratory, thoracic and mediastinal disorders	Rhinorrhoea	70 (1.41)	7.09 (5.6–8.97)	7.05 (363.05)	2.82 (1.15)
Infections and infestations	Influenza	65 (1.31)	3.95 (3.1–5.04)	3.93 (142.24)	1.97 (0.31)
Pregnancy, puerperium and perinatal conditions	Abortion spontaneous	62 (1.25)	10.15 (7.9–13.03)	10.09 (506.84)	3.33 (1.67)
Infections and infestations	Gastroenteritis viral	58 (1.17)	21.46 (16.57–27.8)	21.34 (1119.94)	4.41 (2.74)
Nervous system disorders	Migraine	52 (1.05)	3.64 (2.77–4.78)	3.63 (99.05)	1.86 (0.19)
Respiratory, thoracic and mediastinal disorders	Nasal congestion	51 (1.03)	5.78 (4.39–7.61)	5.76 (200.35)	2.52 (0.86)
Gastrointestinal disorders	Flatulence	46 (0.93)	5.48 (4.1–7.32)	5.46 (167.55)	2.45 (0.78)
Infections and infestations	Ear infection	34 (0.69)	8.52 (6.08–11.93)	8.49 (224.37)	3.08 (1.42)
Infections and infestations	Viral infection	30 (0.61)	6.22 (4.35–8.9)	6.2 (130.85)	2.63 (0.97)
Infections and infestations	Pharyngitis streptococcal	26 (0.52)	15.73 (10.7–23.13)	15.69 (356.62)	3.97 (2.3)
Renal and urinary disorders	Nephrolithiasis	25 (0.50)	3.63 (2.45–5.37)	3.62 (47.44)	1.86 (0.19)
Investigations	Amino acid level decreased	24 (0.48)	536.73 (352.39–817.5)	535.37 (11600.82)	8.92 (7.25)
Infections and infestations	Upper respiratory tract infection	23 (0.46)	3.24 (2.15–4.88)	3.24 (35.55)	1.69 (0.03)

## 4 Discussion

Sapropterin is a synthetic version of tetrahydrobiopterin (BH4), the natural cofactor for the enzyme phenylalanine hydroxylase (PAH). PAH uses an oxidative process to hydroxylate Phe, resulting in tyrosine. In individuals with phenylketonuria, PAH activity is missing or inadequate. Treatment with BH4 can stimulate residual PAH enzyme activity, enhance the natural oxidative metabolism of Phe, and reduce Phe levels in some individuals. It was licensed by the US FDA in December 2007 as the first medication to treat phenylketonuria, a hereditary condition characterized by PAH deficiency. A meta-analysis showed that the most common side effects reported when using sapropterin are abdominal pain, diarrhoea, pyrexia, cough, vomiting, upper respiratory tract infection, headache and oropharyngeal pain (Qu et al., 2019). There were 3 systems that had drug-related events occurring in ≥1% of subjects: gastrointestinal (diarrhea, unspecified gastrointestinal disorder); respiratory (dysphonia and rhinorrhea), and nervous system (amnesia, dizziness, memory impairment, headaches, migraines, poor quality sleep, and psychomotor hyperactivity) (Longo et al., 2015). All of these AEs were detected in our study.

BH4 metabolism plays a key role in neuropsychiatric disorders. Increasing evidence suggests the involvement of peripheral amino acid metabolism in the pathophysiology of neuropsychiatric of disorder. BH4 is a cofactor for enzymes that catalyze phenylalanine metabolism, monoamine synthesis, nitric oxide production, and lipid metabolism (Miyajima et al., 2022). In early treated adults, depressed mood, social isolation/withdrawal, generalized anxiety, and sleep disturbance have been noted (Ashe et al., 2019). Some of these symptoms may be explained by the deficiency of monoamine neurotransmitters. Brain monoamine metabolism was perturbed by the dysfunction in BH4 metabolism. Because BH4 is a highly labile compound, many environmental stresses, such as oxidative stress, infection, and malnutrition, can affect its metabolism. Phenylketonuria is associated with a slight decrease in intelligence (Christ et al., 2010), coupled with impairments in specific aspects of cognition. In particular, individuals with early-treated PKU have difficulty with higher-order cognitive abilities such as planning (Azadi et al., 2009), organization (White et al., 2001), working memory (Channon et al., 2004), and inhibitory control (Christ et al., 2006). At the PT level, our study revealed that although amino acid level increased, anxiety, psychomotor hyperactivity, memory impairment, aggression, abnormal behaviour, irritability, anger, mood altered, disturbance in attention, mood swings, nervousness were significant in the disproportionality analyses, we found that they were also indications for treatment. Therefore, for the accuracy of the study results, we excluded the adverse reactions reported in Table 1 from our results.

This study revealed the AE reporting for sapropterin dihydrochloride from the first quarter of 2008 to the first quarter of 2024. In this study, we found that AEs of sapropterin dihydrochloride occurred more commonly in females (52.55%) than in males (33.60%). The higher number of reports from female patients may reflect the higher prevalence of phenylketonuria in females (Burton et al., 2018). The data show a significant proportion of AEs occurring within 30 days and more than 360 days of medication initiation, highlighting the need for monitoring adverse reactions at the beginning of treatment and long-term follow-up of more than 1 year. It is noteworthy to mention that the majority of adverse event reports are submitted by consumers (79.37%). The country with the most cases reported is the United States (94.71%). This suggests that the remaining countries may lack the emphasis on drug safety and also warns other countries to strengthen monitoring and reporting of adverse reactions. In terms of adverse reaction time, most of the data focused on less than 30 days and more than 360 days, indicating that the occurrence of adverse reactions should be closely monitored at the start of treatment and the use of this drug requires careful long-term follow-up.

The reported AEs with high case reports are consistent with the prescribing information of sapropterin dihydrochloride, such as vomiting, abdominal pain upper and abdominal discomfort. In a 6-week, randomized, placebo-controlled study of sapropterin, the most commonly reported adverse events (occurring at a frequency of ≥10%) were headache (20%), pharyngolaryngeal pain (15%), nasopharyngitis (14%), vomiting (13%), diarrhoea (10%) and upper respiratory infection (10%) (Lee et al., 2008). In an international, double-blind, randomized, placebo-controlled study, the most common adverse events in sapropterin and placebo recipients (occurring in ≥9% of sapropterin recipients) were rhinorrhea (21% and 0%), headache (21% and 8%), cough (15% and 0%), pharynglaryngeal pain (12% and 8%), diarrhoea (12% and 0%), vomiting (12% and 0%) and abdominal pain (9% and 8%) (Trefz et al., 2009). A 26-week open-label, multicentre, randomized phase III b study shows that the most common adverse events classified as related to sapropterin were amino acid concentration decrease [six patients (22.2%)], rhinitis, and vomiting [two patients each (7.4%)], and one patient (3.7%) each for pharyngitis, diarrhea, abdominal pain, mouth ulceration and increased amino acid concentration in patients aged <4 years (Muntau et al., 2017). In the ranking of PT in this study, vomiting (N = 264) ranked first, abdominal pain upper (N = 187) ranked second, and abdominal discomfort (N = 151) ranked third. These complications detected as positive signals are basically consistent with the AEs reported in above clinical trials of sapropterin.

According to the disproportionality analysis, the most significant signals at PT levels was amino acid level decreased, which is a new potential AE. A 3-year extension of the SPARK open-label, multicenter, randomized phase III b trial found that the most commonly reported treatment-emergent adverse event was amino acid level decreased (hypophenylalaninaemia; 24 events occurring in 9 patients) (Muntau et al., 2021). The mechanism underlying the amino acid level decreased with sapropterin may be sapropterin is administered as a replacement for endogenous tetrahydrobiopterin, which is the natural cofactor for the enzyme phenylalanine hydroxylase (Sanford and Keating, 2009).

According to the disproportionality analysis, the most commonly reported signals at SOC levels were gastrointestinal disorders. AEs classified as gastrointestinal disorders at the SOC level included vomiting, abdominal pain upper, dyspepsia, gastrooesophageal reflux disease and flatulence. Among them, vomiting, abdominal pain upper and dyspepsia were recorded in the drug package insert, and the others were newly discovered AEs. The mechanism underlying the increased risk of gastrointestinal disorders with sapropterin is currently unclear but may be related to BH_4_ cofactor levels. In tetrahydrobiopterin deficiency, sapropterin is administered as a replacement for endogenous tetrahydrobiopterin (Sanford and Keating, 2009). BH4 is the cofactor for tyrosine hydroxylase (TH) and the two isoforms of tryptophan hydroxylase (TPH1 and TPH2), the rate limiting enzymes required for the synthesis of the catecholamines (dopamine, norepinephrine, and epinephrine) and serotonin (5HT) (Hyland and Hyland, 2023). The adverse effects of sapropterin on gastrointestinal disorders may be related to the increase of 5HT level through acting on tryptophan hydroxylase, which can activate signals sent to the central nervous system that stimulate digestive reflexes and can cause abdominal pain and discomfort, satiety, or nausea (Mawe and Hoffman, 2013).

According to our literature search, there is no other pharmacovigilance database to study the adverse reactions of sapropterin. In order to better contextualize the findings in relation to the existing body of knowledge, we compared this study with a meta-analysis of sapropterin and sought to unearth possible new insights. In a meta-analysis of randomized controlled trials about sapropterin in patients with phenylketonuria, common adverse events reported in four studies were abdominal pain, diarrhoea, pyrexia, cough, vomiting, upper respiratory tract infection, headache and oropharyngeal pain (Qu et al., 2019). We detected probable new AEs using disproportionality analysis, including gastroesophageal reflux disease, flatulence, influenza, ear infection, viral infection, pharyngitis streptococcal, spontaneous abortion, and nephrolithiasis.

Spontaneous abortion and nephrolithiasis were uncommon new AEs that attracted our attention. Three pregnancies (5.7%) resulted in spontaneous abortions in the first trimester in a US-based, phase 4, observational registry (NCT00778206) (Feillet et al., 2024). Guidelines for the management of phenylalanine hydroxylase deficiency recommend maintenance of maternal blood Phe between 120 and 360 μmol/L. However, median maternal blood Phe < 360 μmol/L was observed in about three-quarters (76.2%, 16/21) of completed pregnancies (N = 17) including the spontaneous abortions (N = 4) in a Maternal Phenylketonuria Observational Program sub-registry (Grange et al., 2014). This means that the decrease of Phe level may not be the cause of spontaneous abortion in pregnant women induced by sapropterin. Sapropterin may cause spontaneous abortion by other means such as endothelial dysfunction which was related to spontaneous abortion by causing defects of the placenta (Pasquier et al., 2013). So far, no reports of renal calculi caused by sapropterin have been found in other literatures. The mechanism of nephrolithiasis induced by sapropterin may be related to its metabolism in the kidney (Bratkovic et al., 2022). Further research is needed to understand the underlying mechanisms linking spontaneous abortion and nephrolithiasis to sapropterin.

It should be noted that among the top 20 PTs ranked by report numbers, the frequency of infections and infestations is second only to gastrointestinal disorders. The reported drugs in FAERS were classified into four modalities: PS (primary suspect), SS (second suspect), C (concomitant), and I (interacting). Because we retained only those reports where sapropterin was listed as the PS drug in the data cleaning phase, we ensured that the AEs of infections and infestations shown were mainly due to sapropterin and not the other combination drugs. The reason for the increased infection may be that the increased BH4 promotes TH to produce more catecholamines (dopamine, norepinephrine, and epinephrine), which are central to multiple complex mechanisms regulating immune function. Neuronal release of norepinephrine directly downregulates inflammatory activity by binding directly to myeloid (Gaskill and Khoshbouei, 2022). The specific mechanism of influenza, ear infection, viral infection, pharyngitis streptococcal, abortion spontaneous, and nephrolithiasis in hyperphenylalaninemia patients treated with sapropterin has not been reported in the literature, and further research is needed.

In order to better understand the difference between the proportion of AEs observed in clinical trials and the real-world data from the FAERS database, the proportion of common AEs in the real-world data were calculated and compared to clinical trials. In a 6-week, randomized, placebo-controlled study of sapropterin, AEs that were considered to be probably related to sapropterin were vomiting (4 patients, 12.90%), diarrhea (2 patients, 6.45%) and abdominal pain (2 patients, 6.45%) in 31 patients (Lee et al., 2008). In an international, double-blind, randomized, placebo-controlled study, vomiting (4 patients, 12.12%), diarrhea (4 patients, 12.12%) and abdominal pain (3 patients, 9.09%) in 33 patients of the sapropterin group were observed (Trefz et al., 2009). In Table 4, we could find that these real-world data [vomiting (5.33%), abdominal discomfort (3.05%), abdominal pain upper (3.78%)] suggest a lower proportion compared to clinical trial data. These findings are expected because the sample size of clinical trials is small (nearly 30 patients), especially it is difficult to find patients with rare diseases who meet the test conditions. This means that the proportion of some common adverse reactions might be amplified and rare adverse reactions can be observed with very low probability in clinical trials. However, real-world data based on FAERS database could more objectively capture the occurrence of AEs due to its large sample size (4,953 patients), which reflects the necessity and advantage of using real data based on Fares database to observe adverse reactions. Since the subjects of the clinical trial data cited were patients with PKU and hyperphenylalaninemia, it was impossible to compare the differences in adverse effects of the drug between patients and healthy people.

At the same time, since most of the data on adverse events of sapropterin presented in the FAERS database came from the United States, we also sought to find reports on AEs in other countries and compare the results with those in this paper. A Japanese post-marketing surveillance study showed that only one patient (1.2%) experienced an increase in alanine aminotransferase level in 85 patients <65 years and the occurrence of any other adverse events related to sapropterin was not observed (Tamura et al., 2022). A multicenter phase III b study in 9 countries [Austria (n = 2), Belgium (n = 2), Czech Republic (n = 1), Germany (n = 4), Italy (n = 5), Netherlands (n = 2), Slovakia (n = 3), Turkey (n = 1) and the United Kingdom (n = 2)] showed that amino acid concentration decrease (6 patients, 22.2%), rhinitis (2 patients, 7.4%), vomiting (2 patients, 7.4%), pharyngitis (1 patients, 3.7%), diarrhea (1 patients, 3.7%), abdominal pain (1 patients, 3.7%), mouth ulceration (1 patients, 3.7%) and increased amino acid concentration (1 patients, 3.7%) were associated with the use of sapropterin in children <4 years with confirmed BH4-responsive phenylketonuria or mild hyperphenylalaninemia (Muntau et al., 2017). We found that vomiting may be a relatively uncommon adverse reaction in other countries while it was relatively common in United States based on FAERS database. The emergence of amino acid level decreased in the monitoring of AEs with sapropterin in other countries may be more pronounced. The differences may be due to country, age, or sample size. Subsequent surveillance of AEs with larger samples in other countries is needed for further analysis.

This study gave in-depth insights on AEs following the usage of sapropterin by evaluating the FAERS database. However, the FAERS database also has certain shortcomings. Firstly, the data in the FAERS database are spontaneously provided by healthcare workers, consumers, physicians and others, with different data quality, correctness, and completeness. Reporting bias, underreporting, and incomplete data can result in an overrepresentation of certain adverse events while underestimating others, particularly in regions or countries with less robust reporting mechanisms. In this study, we used the Medical Subject Headings (MeSH) thesaurus (https://www.ncbi.nlm.nih.gov/mesh) to search all the drug nomenclature of sapropterin including medication brand, trade names, generic names and non-proprietary names. The MeSH thesaurus is a controlled and hierarchically-organized vocabulary produced by the National Library of Medicine. This ensured that the data we collected were complete enough to mitigate the effects of missing data. In addition, we retained only those reports where sapropterin was listed as the PS drug to reduce variability in report quality. Secondly, the data in the FAERS database predominantly originate from American populations (94.71%), with comparatively few reported data from other groups. It should be noted that different populations in different countries may have different sensitivity to sapropterin, resulting in different AEs effect. Thirdly, the data in the FAERS database are based on observational reports, which is not feasible to directly prove causation from these data alone and is a frequent flaw in all pharmacovigilance research. The AEs reported might be connected to sapropterin or may be due to other reasons. For specification, we retained only those reports where sapropterin was listed as the PS drug, which could reduce the suspicion that AEs were due to other combination drugs. Finally, the reliance on spontaneous reporting to the FAERS database introduces potential biases, including underreporting or selective reporting of severe AEs in report quality. In the descriptive report, the vast majority of reports were provided by consumer (79.37%). Since the medical professional degree of users was not as professional as that of medical personnel, different customers would have different reporting standards, resulting in different quality of reporting results. Based on subjective judgment of AEs, they might capture some common AEs, such as gastrointestinal reactions, resulting in over-expression, while some rare AEs were difficult to describe accurately. In addition, the underreporting of some serious AEs by patient groups may be due to their poor awareness of the reporting system (Al Dweik et al., 2017). These would affect our ability to accurately capture new adverse effects. These factors necessitate cautious interpretation of our findings and underscore the need for continuous and multifaceted pharmacovigilance efforts.

Additionally, the study did not contain extensive clinical analysis, such as case-by-case evaluations or co-reported medication analyses. Future research should combine these extensive analyses to offer more comprehensive insights into the safety profile of sapropterin. Based on the results of this study, clinicians who prescribe sapropterin for PKU or hyperphenylalaninemia patients should closely monitor the possible AEs for a long time. For pregnant women, the possibility of spontaneous abortion after taking sapropterin should be closely observed, although the possibility of its occurrence is extremely low. In addition, patients with concomitant infections and nephrolithiasis should be closely monitored for sapropterin to aggravate these symptoms. When these serious AEs occur, the drug should be stopped in time and the risk of continuing the drug should be evaluated after the adverse reactions disappear. The newly discovered AEs associated with sapropterin highlighted in this article can assist clinicians in maintaining heightened vigilance when prescribing sapropterin, which can reduce the occurrence of risks through rational interventions.

## 5 Conclusion

Sapropterin, being first medicine used to PKU treatment, has prompted concerns regarding its safety in the medical community. This study was based on the FAERS database in the United States and conducted a comprehensive analysis of AEs following the use of sapropterin, uncovering a range of known and potential AEs. Although sapropterin has shown efficacy in treating phenylketonuria in clinical trials, we have identified several potential AEs, such as nasopharyngitis, gastrooesophageal reflux disease, influenza, ear infection, viral infection, pharyngitis streptococcal, abortion spontaneous and nephrolithiasis. These findings underscore the importance of meticulous patient monitoring and the necessity for individualized treatment plans while using sapropterin. Furthermore, the findings of this study provide directions for future research, where exploring the specific mechanisms and management strategies related to sapropterin-associated AEs will be crucial for enhancing patient treatment safety and efficacy.

## Data Availability

Publicly available datasets were analyzed in this study. This data can be found here: https://fis.fda.gov/extensions/FPD-QDE-FAERS/FPD-QDE-FAERS.html.

## Abbreviations

AEs, Adverse events; FAERS, FDA Adverse event reporting system; PTs, Preferred terms; SOC, System organ class; ROR, Reporting odds ratio; PRR, Proportional reporting ratio; BCPNN, Bayesian confidence propagation neural network; PKU, Phenylketonuria; BH4, Tetrahydrobiopterin; PAH, Enzyme phenylalanine hydroxylase; Phe, Phenylalanine; HPA, Hyperphenylalaninemia.
