# Supplementary material for: Safety assessment of sapropterin dihydrochloride: real-world adverse event analysis based on the FDA adverse event reporting system (FAERS)
Source: Front Pharmacol. 2024 Oct 25;15:1486597. doi: 10.3389/fphar.2024.1486597 (PMC11543418; doi:10.3389/fphar.2024.1486597)
Supplement: Supplementary file 1 [file Table1.DOCX]

Supplementary Material

# Supplementary Tables

Supplementary Table 1: The top 20 signal strengths of adverse events associated with sapropterin dihydrochloride ranked by ROR at the PTs level in the FAERS database.

| SOC | PTs | Case Reports | ROR(95% CI) | PRR(χ^2^) | IC(IC025) |
| --- | --- | --- | --- | --- | --- |
| Investigations | Amino acid level decreased | 24 | 536.73 ( 352.39 - 817.5 ) | 535.37 ( 11600.82 ) | 8.92 ( 7.25 ) |
| Gastrointestinal disorders | Teething | 4 | 95 ( 35.33 - 255.45 ) | 94.96 ( 365.18 ) | 6.54 ( 4.87 ) |
| Musculoskeletal and connective tissue disorders | Growth accelerated | 3 | 93.56 ( 29.86 - 293.11 ) | 93.53 ( 269.74 ) | 6.52 ( 4.84 ) |
| Infections and infestations | Hand-foot-and-mouth disease | 6 | 59.64 ( 26.66 - 133.39 ) | 59.6 ( 341.76 ) | 5.88 ( 4.21 ) |
| Skin and subcutaneous tissue disorders | Dermatitis diaper | 6 | 57.97 ( 25.92 - 129.64 ) | 57.93 ( 331.98 ) | 5.84 ( 4.17 ) |
| Pregnancy, puerperium and perinatal conditions | Hyperemesis gravidarum | 3 | 50.75 ( 16.28 - 158.27 ) | 50.74 ( 144.85 ) | 5.65 ( 3.98 ) |
| Congenital, familial and genetic disorders | Microcephaly | 8 | 42.76 ( 21.32 - 85.78 ) | 42.73 ( 323.33 ) | 5.41 ( 3.74 ) |
| Pregnancy, puerperium and perinatal conditions | Morning sickness | 5 | 36.06 ( 14.96 - 86.92 ) | 36.04 ( 169.16 ) | 5.16 ( 3.49 ) |
| Infections and infestations | Croup infectious | 3 | 26.96 ( 8.67 - 83.86 ) | 26.95 ( 74.59 ) | 4.75 ( 3.07 ) |
| Infections and infestations | Gastroenteritis viral | 58 | 21.46 ( 16.57 - 27.8 ) | 21.34 ( 1119.94 ) | 4.41 ( 2.74 ) |
| Infections and infestations | Gastrointestinal viral infection | 3 | 18.27 ( 5.88 - 56.77 ) | 18.27 ( 48.79 ) | 4.19 ( 2.52 ) |
| Skin and subcutaneous tissue disorders | Yellow skin | 13 | 17.28 ( 10.02 - 29.79 ) | 17.26 ( 198.42 ) | 4.1 ( 2.44 ) |
| Infections and infestations | Pharyngitis streptococcal | 26 | 15.73 ( 10.7 - 23.13 ) | 15.69 ( 356.62 ) | 3.97 ( 2.3 ) |
| Ear and labyrinth disorders | Middle ear effusion | 5 | 14.84 ( 6.17 - 35.7 ) | 14.83 ( 64.3 ) | 3.89 ( 2.22 ) |
| Infections and infestations | H1n1 influenza | 3 | 12.36 ( 3.98 - 38.4 ) | 12.36 ( 31.25 ) | 3.62 ( 1.96 ) |
| Pregnancy, puerperium and perinatal conditions | Foetal growth restriction | 11 | 10.78 ( 5.97 - 19.49 ) | 10.77 ( 97.32 ) | 3.43 ( 1.76 ) |
| Respiratory, thoracic and mediastinal disorders | Neonatal respiratory distress syndrome | 6 | 10.73 ( 4.81 - 23.91 ) | 10.72 ( 52.79 ) | 3.42 ( 1.75 ) |
| Pregnancy, puerperium and perinatal conditions | Abortion spontaneous | 62 | 10.15 ( 7.9 - 13.03 ) | 10.09 ( 506.84 ) | 3.33 ( 1.67 ) |
| Pregnancy, puerperium and perinatal conditions | Foetal distress syndrome | 3 | 9.56 ( 3.08 - 29.67 ) | 9.55 ( 22.94 ) | 3.25 ( 1.59 ) |
| Psychiatric disorders | Logorrhoea | 5 | 9.17 ( 3.81 - 22.05 ) | 9.16 ( 36.3 ) | 3.19 ( 1.53 ) |
